# Plasticity in nesting adaptations of a tidal marsh endemic bird

**DOI:** 10.1002/ece3.4528

**Published:** 2018-10-18

**Authors:** Bri Benvenuti, Jennifer Walsh, Kathleen M. O'Brien, Adrienne I. Kovach

**Affiliations:** ^1^ Department of Natural Resources and the Environment University of New Hampshire Durham New Hampshire; ^2^ Rachel Carson National Wildlife Refuge United States Fish and Wildlife Service Wells Maine; ^3^ Present address: Fuller Evolutionary Biology Program Cornell Laboratory of Ornithology Ithaca New York

**Keywords:** nest site selection, plasticity, renesting, saltmarsh sparrow, site fidelity, tidal marsh

## Abstract

If individuals can perceive and manage risks, they may alter their behaviors based on prior experience. This expectation may apply to nest site selection of breeding birds, for which adaptive behavioral responses may enhance fitness. Birds that nest in tidal marshes have adapted to the challenges posed primarily by periodic, monthly tidal flooding and secondarily by predation. We investigated adaptive responses in nesting behavior of the saltmarsh sparrow (*Ammospiza caudacutus*), an obligate tidal‐marsh‐breeding bird, using 536 nests monitored across 5 years. Using linear mixed effects models, we tested whether nest characteristics differed among nests that were successful, depredated, or flooded, and we investigated whether females made changes in nest structure and placement according to outcome of their previous nesting attempt. Nest characteristics differed among females with different nest fates. Fledged and depredated nests were built higher in the vegetation and in higher elevation areas of the marsh than those that flooded. Successful nests had more canopy cover and were comprised of a lower proportion of high marsh vegetation (*Spartina patens*) than those that were flooded or depredated. Females with nests that failed due to flooding constructed subsequent nests higher in the vegetation and at higher elevation than those that were successful in their prior attempt, consistent with a response to previous experience. Eighty‐five percent of females renested within the average home range core area distance (77 m), indicating a high degree of nest placement fidelity. Females for which nests were depredated in their prior nesting attempt renested at a greater distance than females for which the previous nesting attempts were successful. Our findings suggest saltmarsh sparrows exhibit plasticity in nesting behavior, which may be important for balancing selective pressures in a dynamic environment. This plasticity, however, is insufficient to enable them to adapt to the increased flooding predicted with sea‐level rise.

## 1 INTRODUCTION

Nest site selection in birds should be such that it enhances the survival and fitness of offspring, as well as the reproductive success of the parent(s) (Lovich et al., 2014; Shine & Harlow, 1996). Accordingly, individuals must balance nest placement, weighing requirements for survival and risks (Hanane, 2014). Associating specific nest site attributes with nesting success may be a learned adaptation (Gavin & Bollinger, 1988; Marzluff, 1988), and it may be one method of mitigating risks of nest failure. If breeding females can both perceive and manage risks, they may be expected to alter their nesting behaviors based on prior experience. For example, in areas of high nest predation, it may be adaptive to move away from risky sites or make alterations in nest structure to reduce the risk of failure (Beckmann, Biro, & Martin, 2015; McAuley, Longcore, & Sepik, 1990). Conversely, by exhibiting fidelity to the same breeding location yearly, birds may gain advantages that are positively correlated with breeding success, such as knowledge of food availability and predator densities (Chalfoun & Schmidt, 2012). Adaptive responses to multiple environmental factors require that a female learns specific nest site attributes and their vulnerability to specific environmental factors (Marzluff, 1988). Multiple studies on nest site selection in diverse species have found that individuals will use information on their previous breeding success to choose a current breeding site (Gavin & Bollinger, 1988; McAuley et al., 1990; Beletsky & Orians, 1991; Haas, 1998; Hunter et al. 2016). This informed nest site fidelity combined with plasticity in nest structure could lead to greater reproductive success (Chalfoun & Schmidt, 2012; Switzer, 1997).

Nest site selection is directly correlated with reproductive success in tidal marsh nesting birds, which experience high levels of nest failure due to tidal flooding (Gjerdrum, Elphick, & Rubega, 2005; Storey, Montevecchi, Andrews, & Sims, 1988). In tidal marshes, water levels fluctuate predictably with the 28‐day lunar cycle, producing peaks in tide height with the new and full moons and resulting in one to two consecutive days each cycle when marshes are flooded almost entirely (Redfield, 1972). Tidal marsh specialists have adapted to the challenges of living in this harsh environment, with the trade‐off being limited interspecific competition and abundant resources (Greenberg et al., 2006; Reinert, 2006). Birds that nest in tidal marshes exhibit some adaptations to mitigate reproductive consequences of tidal nest flooding, such as placement of nests at a height that exceeds the tides but is low enough to the marsh surface to minimize predation, nest repair or egg retrieval behaviors, rapid postflood renesting, and timing of nesting attempts to avoid peak seasonal tides (Greenberg et al., 2006; Reinert, 2006). Given the strong selection pressure imposed upon tidal marsh birds by periodic tidal flooding, and variation in the relative risks of predation and flooding both within and across marshes (e.g., Hunter et al., 2016, Ruskin et al., 2017a), it may also be adaptive for them to assess risks and exhibit plasticity in nesting behavior (Forstmeier & Weiss, 2004).

**Figure 1:**
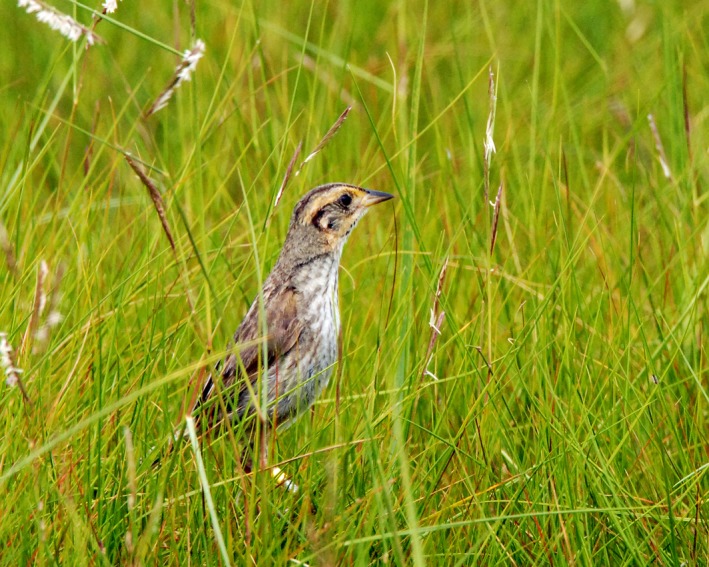
Adult saltmarsh sparrow in *Spartina patens*. Photo courtesy of Nancy J. Landry

**Figure 2:**
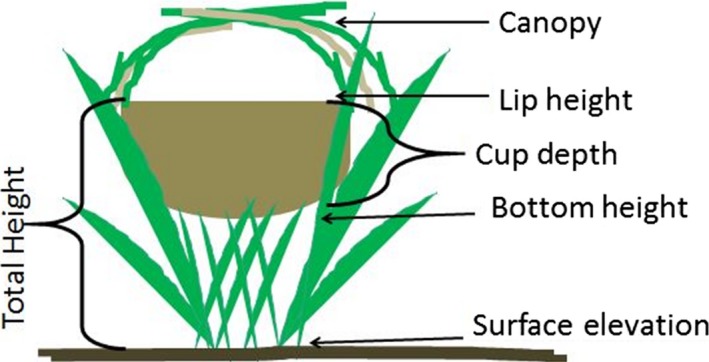
Illustration of saltmarsh sparrow nest, woven into marsh vegetation, showing structural features of the nest in relation to the marsh surface. For nests to escape flooding, tidal water levels will rise above the marsh surface, but will not exceed the height of the nest. The canopy may help to retain eggs in a flooded nest

The saltmarsh sparrow (*Ammospiza caudacutus*; Figure 1) is a tidal marsh specialist with reproductive behaviors shaped by the unique selective pressures of tidal marshes. The breeding system is highly promiscuous and is characterized by scramble competition among males for mating access (Hill, Gjerdrum, & Elphick, 2010; Walsh, Maxwell, & Kovach, 2018). Neither sex is territorial and males provide no parental care (Post & Greenlaw, 1982). Females build ground nests a few centimeters above the surface of the marsh, just above the height of the mean high water level (Gjerdrum et al., 2005; Shriver, Vickery, Hodgman, & Gibbs, 2007; see Figure 2). Nests that are initiated within three days of a high spring tide are most likely to be successful by avoiding peak tidal flooding that occurs with the 28‐day lunar cycle (Gjerdrum et al., 2005; Greenlaw & Rising, 1994; Shriver et al., 2007). Nests are woven into the vegetation—primarily *Spartina patens*,* S. alterniflora*, and *Juncus gerardii*—and located in areas of higher elevation within the marsh, which are less affected by tidal flooding (Gjerdrum et al., 2005; Shriver et al., 2007). Nest site selection is spatially random with respect to other nesting females (Bayard & Elphick, 2010; Gjerdrum et al., 2005), suggesting that structural characteristics of the nest itself may be more important to success than where the nest is located within the preferred nesting habitat (Gjerdrum et al., 2005). While prior research has found vegetation cover characteristics to be important in nest site selection, neither these vegetation characteristics, nor nest height (the height at which the nest is placed above the ground) and substrate elevation (the elevation of the marsh surface; see Figure 2) have been found to consistently influence nest success (Gjerdrum et al., 2005; Humphreys, Elphick, Gjerdrum, & Rubega, 2007; Ruskin, Hodgman, Etterson, & Olsen, 2015; Shriver et al., 2007).

As for other tidal‐marsh‐breeding birds, nest failure of saltmarsh sparrows may be a result of either flooding or depredation by avian or mammalian predators, suggesting there is a trade‐off between flooding and predation risks that influence nesting behaviors (Greenberg et al., 2006; Ruskin et al., 2017a). By nesting higher in the vegetation, a female's nest is more visible and susceptible to predation, while nesting closer to the marsh surface will increase the likelihood of nest flooding. Further, females may construct a canopy above the nest, which can function in retaining eggs during flooding events and may reduce predation through additional vegetation cover and concealment (Humphreys et al., 2007). A lack of territoriality, pair‐bonding, or paternal care (Greenlaw & Rising, 1994; Hill et al., 2010) suggests that nest locations may be labile and females may potentially renest in new locations during the breeding season in response to nesting failure. Whether females can perceive the mortality risks facing their nests, as well as the characteristics that are associated with these risks, and alter their nesting behaviors in response is unknown.

We investigated characteristics of female nest site selection and sought to determine whether females modified their nesting behaviors as a function of prior experience. We collected data on nest characteristics, including location, marsh elevation, and structural features, to address the following questions:

1. Do nest characteristics differ among nests that are successful and those that fail due to flooding or predation? We sought to investigate differences in nest site elevation, nest height, canopy presence, and vegetation composition among nests and compared them with ultimate fates of fledged, flooded, and depredated. We predicted successful nests would be in areas of higher elevation and have characteristics that simultaneously minimize the effects of predation and flooding (e.g., presence of canopy cover, nest height above tidal flooding level, and vegetation composition primarily of *S. patens*).
2. Do female saltmarsh sparrows exhibit nest placement fidelity across years? We aimed to determine whether female saltmarsh sparrows returned to the same locations to nest in future years based on the fate of their previous year's nesting attempt. We hypothesized females would renest within their prior home range core area across subsequent years due to the advantages of local resource knowledge, such as fine‐scale vegetation and elevation patterns.
3. Do females make changes in their nest site location and structure based on previous experiences and the outcomes of their prior nesting attempts—whether it failed due to predation or flooding or was successful? We sought to explore changes in nest placement and structure relative to a female's prior nesting success. We expected females to alter the location, elevation, and structural characteristics of their nests in a way that would increase nesting success relative to the outcome of their prior nesting attempt. We predicted that, within a breeding season, females whose nests failed due to flooding would make structural changes to subsequent nests that would mitigate flooding failure, such as an increase in nest height, canopy cover, changes in vegetation composition, or renest in a higher elevation area of the marsh. Furthermore, we predicted that females whose nests failed due to predation would renest at a greater distance from their previous nest, rather than modifying structural characteristics.

## 2 METHODS

### 2.1 Study area

**Figure 3:**
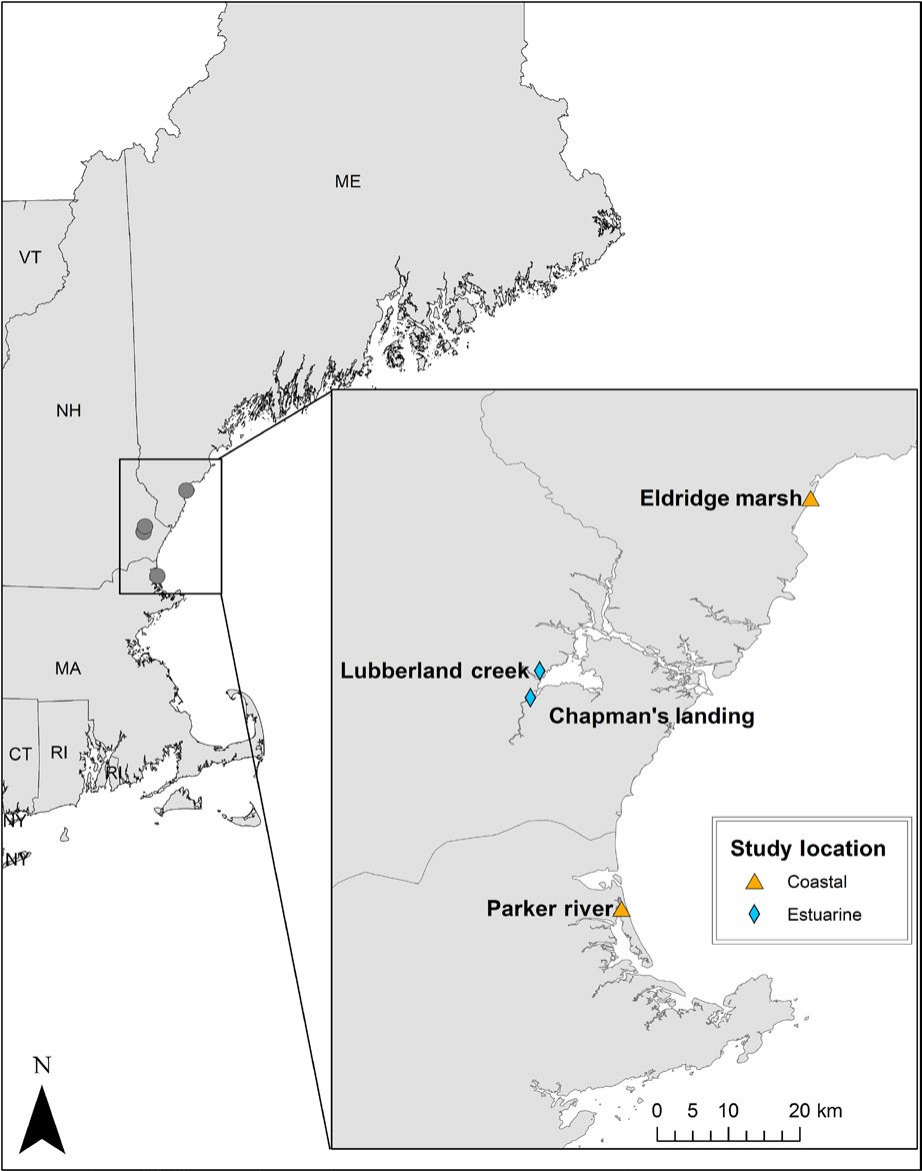
Locations of the four marshes in the northeastern United States where saltmarsh sparrows nesting data were collected during 2011–2015

We conducted intensive monitoring of saltmarsh sparrow nests on four tidal marshes in the northeastern United States during the breeding season (June–August) from 2011 to 2015. All work was conducted in accordance with protocols approved by the Institutional Animal Care and Use Committee of the University of New Hampshire (nos. 100605 and 130604). Study sites were located in Stratham, New Hampshire (43.04N 70.72W; Chapman's Landing), Newmarket, New Hampshire (43.07N 70.91W; Lubberland Creek Preserve), Wells, Maine (43.29N 70.57W; Eldridge Marsh, Rachel Carson National Wildlife Refuge [NWR]), and Newburyport, Massachusetts (42.77N, 70.80W; Parker River NWR; Figure 3). The area monitored on each site varied from 10 to 18 ha. On Chapman's Landing and Lubberland Creek (11 and 10.5 ha, respectively) the study site included the entire marsh. On the two larger marshes, Parker River and Eldridge Marsh, we focused on 18 ha and 15 ha plots, respectively. The sites differed by proximity to the coast and tidal regime: Chapman's Landing and Lubberland Creek were located farther inland within the Great Bay estuary, with a tidal amplitude of 2.7 m, while Eldridge Marsh and Parker River were coastal marshes with a tidal amplitude of 3.3 m.

### 2.2 Nest placement and monitoring

Sites were systematically searched for nests 2–3 times per week during each of the three annual nesting cycles. Once found, nests were revisited every 3–4 days until the nesting attempt was completed. Nests were assigned one of three ultimate nest fates: fledged, failure due to flooding, or failure due to depredation, following Ruskin et al. (2017a,b). Nests were considered fledged if one individual from the nest reached fledging age (i.e., nests could experience partial failure prior to fledging). Nests were considered to have failed due to flooding if one or more eggs or nestlings were found immediately outside of the nest cup or the nest contents were cold and wet with the female no longer attending the nest. Nests were deemed depredated when there were signs of predatory activity, such as disturbed nests or partial remains of nestlings, and none of the chicks fledged.

Immediately upon finding a nest, we recorded structural nest measurements, including nest height (lip to ground and bottom to ground), canopy presence (coded as a categorical variable with 3 levels: full, partial, or absent), canopy cover (percentage of nest cup visible from above), and cup depth (Ruskin et al., 2017a,b; see Figure 2). Species vegetation composition at the nest was collected upon nest completion to minimize disturbance to active nests and surrounding vegetation. Vegetation composition was recorded as percentage of high marsh vegetation. *Spartina patens*,* Juncus gerardii*, and *Distichlis spicata* were considered high marsh habitat, while bare ground, open water, and *Spartina alterniflora* (both tall and short form; Gallagher, Somers, Grant, & Seliskar, 1988) were considered low marsh habitat (Roberts et al., 2017). For testing associations of nest characteristics with nest fate, we used data from 536 nests monitored across the 5‐year study (see below for statistical analysis methods).

In 2015, we used a Trimble TSC3 data logger with real‐time kinematic (RTK) R10 Glonass‐enabled antenna (Trimble Navigation Limited, Sunnyvale, CA) and CORS base station correction (Keystone Precision, Durham, NH) to determine the surface elevation (see Figure 2) at found nests. Using these methods, we collected elevation data from 120 nests (out of the total of 536 across the 5‐year study), including 12 females with >1 nesting attempt, to test for elevational influences on nest fate and changes in nest elevation over repeat nesting attempts. Because the total height of a nest above the tidal water level is a function of both the marsh surface elevation at the nest site and the height of the nest in the vegetation, we also considered a new parameter—total nest height (surface elevation + bottom nest height). We calculated total nest height by adding the marsh surface elevation at the nest to the bottom nest height. Due to a small sample of females with multiple nesting attempts for which the first nest was depredated (*n* = 14), we used only females with prior nest fates of fledged (*n* = 74) and flooded (*n* = 32) to investigate changes in marsh elevation and total nest height.

To track multiple nesting attempts from the same individual throughout the breeding season and across years, females attending nests were captured at the nest using two 12‐m, 36‐mm mesh mist nets and uniquely marked with a numbered USGS aluminum leg band. Nest locations were recorded using a GPS unit (Garmin GPSmap 76Cx). To address hypotheses related to female nest placement across years, we used only nesting attempts from consecutive years (*n* = 78 females). For hypotheses about nest movement within a breeding season, we used all females with multiple nesting attempts within that breeding season (*n* = 45 females); however, we could not always be certain the nesting attempts were immediately sequential as there may have been intervening nesting attempts that were not found. We used GENALEX 6.5 (Peakall & Smouse, 2012) to calculate the Euclidean distance between nest locations for a given female within and across breeding seasons.

### 2.3 Statistical analyses

To test our hypothesis that nest site selection and characteristics differed among nests of different fates, we compared nest site elevation and structural characteristics among nests of the three ultimate fates of fledged, flooded, and depredated. To address any potential confounding effects of nest discovery bias, that is failed nests are available for discovery for fewer days than successful nests (Mayfield 1961), we first examined our data for differences in initial discovery date for nests of different fates. Most nests were found early in the nesting cycle (mean = 8.5 days) although there were slight differences in the initial discovery dates across fates (*GLMM,* χ^2^ *=* 14.44*, p *<* *0.001). On average, flooded (7.5 days) and predated (8.1 days) nests were found 1–2 days earlier than fledged nests (9.9 days). Given that the magnitude of this difference was small and in the opposite direction of potential concern—that is, failed nests are available for discovery for fewer days than successful nests, yet we found them slightly earlier than successful nests—and that we had a very intensive nest sampling effort, we think it is reasonable to assume that discovery bias is not of concern in our study and it is appropriate to make inferences about the three nest fate categories.

We tested for differences in nest structural characteristics (bottom nest height, lip nest height, cup depth, canopy presence and cover, and vegetation composition) across the three fate categories (fledged, flooded, and depredated) using GLMMs with assumed normal errors in the package “nlme” (Pinheiro et al. 2016) in R statistical software (R Development Core Team, 2014). We tested for differences in the structural characteristics among sites (the four different study marshes) and locations (inland vs. coastal sites), using ANOVA. Nest height and canopy presence varied significantly among locations (*p* < 0.001), and we therefore included location as a fixed effect covariate for mixed models with those two factors. We also incorporated female identity as a random effect to account for variation among females. To test for differences in each structural characteristic among fates, we used post hoc Tukey's highly significant difference tests for pairwise comparisons in the R package “multcomp” (Hothorn, Bretz, & Westfall, 2008). Description of all models and covariates is found in Appendix 2.

We tested for site‐specific differences in marsh surface elevation using an ANOVA and Tukey's highly significant difference tests for pairwise differences. We found a significant difference in site elevation for all pairwise combinations except Eldridge Marsh and Parker River; therefore, we included site as a covariate in subsequent analyses. We then tested for differences in elevation at the nest site among nests of the three fate categories, using a GLMM with nest fate as a fixed effect, female identity as a random effect, and nest elevation as the response variable. Similarly, we also tested for differences in total nest height (surface elevation (cm) + bottom nest height (cm)) across the three fates.

Next, we examined whether sparrows made changes in nest structure in response to their previous nest outcome. We used GLMMs with a random effect of female identity, fixed effect of prior nest fate, and response variable of difference in nest characteristic measurement to test for changes in nest structure between nest attempts as a function of nest fate. For these tests, we only used the numerical measurements (nest height, cup depth, vegetation composition, percent visible), and we calculated the difference between measurements for the first and second nesting attempts.

To determine whether females moved to areas of higher elevation in renesting attempts following a flooding failure versus success, we used a GLMM with change in elevation (elevation nest 2 – elevation nest 1) as the response variable and prior nest fate as a fixed effect. Similarly, we tested for changes in total nest height by comparing total height nest 2 – total height nest 1 across the three initial nest fates. We assessed the significance of our fixed effect of fate using F tests and type II sums of squares. Description of all models and covariates for this set of analyses is found in Appendix 3.

It is possible that a relationship in changes in nesting behavior and prior fate could arise from a statistical artifact resulting from regression to the mean, due to the bounded nature of the nest data (i.e., there are minimums and maximums for all measured characteristics; Bailey et al., 2017). To rule out this possibility for any significant observed relationships, we quantified the relationship between changes in nesting behavior and prior nest fate in randomized data, following Bailey et al. (2017). To do so, we first randomized the nest order with 5,000 iterations using MATLAB. We then calculated the change in nest behavioral measurement for the randomized data and ran the same model test for statistical associations. We plotted the distribution of *p*‐values for the randomly generated data and calculated the percentage of models with *p* < 0.05.

Lastly, we examined whether female nest site fidelity was influenced by prior nest fate. We used GLMMs with assumed normal errors to test for a relationship of nest fate and distance moved by females between nesting attempts. Models included distance between nesting attempts as the response variable, fate of the prior nesting attempt as a fixed effect, and female identity as a random effect for both within‐year and across‐year comparisons. Significance was assessed using *F* tests and type II sums of squares. To test whether females showed nest placement fidelity within and across years, we used a one‐sided *t* test to determine whether the mean distance moved between nesting attempts was greater than the average diameter of the home range core area of female saltmarsh sparrows (77 ± 13 m; Shriver, Hodgman, Gibbs, & Vickery, 2010).

## 3 RESULTS

We located and monitored 556 nests across the four study sites from 2011 to 2015. We assigned fates to 536 nests, of those 393 (73%) also had information on the attending female. Sample sizes and female capture rates per site and year are in Appendix 1. Only 30 (5%) nests experienced partial depredation and partial flooding (1 partial depredation and 29 partial flooding). We removed the single partially depredated nest from further analyses. Nests that experienced partial flooding were similar in structure to nests that failed due to flooding, and we therefore included them within the flooded fate category (results were the same for analyses conducted with and without the partially depredated nests). We obtained renesting data (1–5 repeat nesting events) for 98 pairs of nests from 78 individual females within a single year and for 57 pairs of nests for 45 females across years; these were the sample sizes used in the nest fidelity analyses. Of these renesting attempts, we had complete data on nesting characteristics and fate from 64 females for 84 pairs of nests (some females had >1 renesting attempt) used in models assessing changes in nest structure and location as a function of prior experience (nest fate).

### 3.1 Do nest characteristics differ among fledged, flooded, and depredated nests?

**Figure 4:**
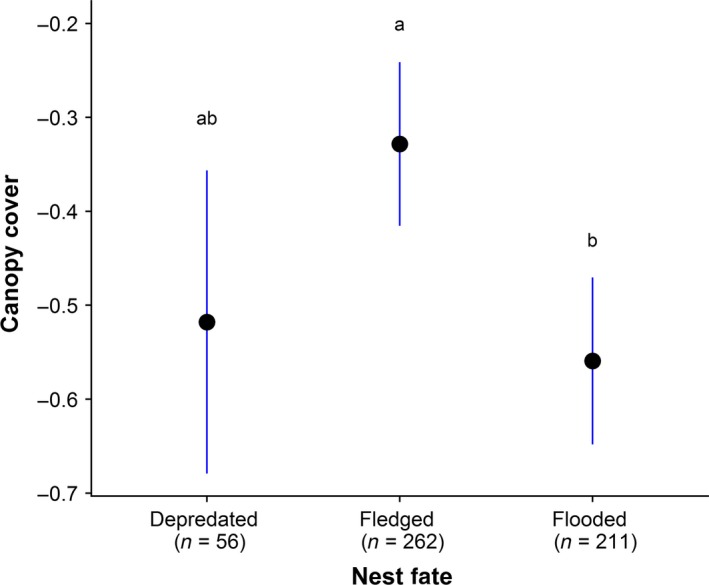
Comparison of the extent of canopy cover among the three nest fates—predated, fledged, and flooded. Canopy cover was assessed for each nest according to three categories: 1 = no canopy, 0 = partial canopy, and 1 = full canopy. Mean values closer to 0 indicate a greater proportion of canopy cover while values closer to −1 indicate little to no canopy cover

Several nest characteristics differed among fledged, flooded, and depredated nests (*n* = 536; Table 1; Appendix 2). Nest height (bottom to ground) differed across nest fates (*GLMM,* χ^2^ = 28.06*, p *<* *0.001): Successful nests were built higher than flooded nests (*Tukey*,* z *=* *−3.98, *p < *0.001), and flooded nests were constructed lower than depredated nests (*Tukey*,* z *=* *−4.51*, p < *0.001). Nest height (lip to ground) was higher in successful nests than flooded nests (*Tukey*,* z *=* *−4.06*, p < *0.001). Nest height (lip to ground) also differed significantly between flooded and depredated nests (*Tukey*,* z *=* *−4.69, *p < *0.001), with depredated nests highest of all (*GLMM,* χ^2^ = 29.11*, p *<* *0.001). There was no difference in the height (measured as either bottom or lip to ground) of successful and depredated nests. The presence of a canopy differed across fates (*GLMM,* χ^2^ = 10.11*, p *=* *0.006; Figure 4), with fledged nests more likely to have full or partial canopy than flooded nests (*Tukey*,* z *=* *−3.18*, p = *0.004); there was no significant difference in canopy presence between depredated and flooded nests or between fledged and depredated nests, although the trend was for less canopy presence in the latter. The proportion of high marsh vegetation also differed by nest fate (*GLMM,* χ^*2*^ = 6.81*, p *=* *0.03). Depredated nests had the greatest proportion of high marsh vegetation, followed by flooded nests, and fledged nests had the least amount of high marsh vegetation, although post hoc Tukey's tests for pairwise differences were not significant. We found no differences in the percentage of the nest visible or nest cup depth across fates.

**Table 1:** Mean nest characteristic measurement ± *SE* for 536 saltmarsh sparrow nests in each of the three fate categories—flooded, fledged, and depredated. Results of Tukey HSD comparisons are provided, with different letters indicating statistically different comparisons

	Flooded (*n* = 223)		Fledged (*n* = 271)		Depredated (*n* = 61)	
Bottom nest height (cm)	10.3 ± 0.3	A	12.3 ± 0.3	B	13.4 ± 0.7	B
Lip nest height (cm)	16.5 ± 0.3	A	18.5 ± 0.3	B	20.0 ± 0.8	B
High marsh (%)	56.3 ± 2.1	A	51.0 ± 1.7	A	59.2 ± 4.2	A
Canopy cover (%)^b^	24.1 ± 1.9	A	23.2 ± 1.6	A	23.1 ± 3.7	A
Cup depth (cm)	6.1 ± 0.2	A	6.2 ± 0.1	A	6.7 ± 0.4	A
Surface elevation (cm)^a^	133.4 ± 3.0	A	138.3 ± 2.0	B	–	NA
Total height (cm)	143.9 ± 3.1	A	149.8 ± 2.1	B	–	NA

a Data for elevation and total height were only collected in one of 5 years (sample sizes: fledged, *n* = 74; flooded, *n* = 32).

b Canopy presence was also examined with categorical values; see Figure 4.

Surface elevation, as measured by RTK data in 2015, differed between all pairs of sites except Eldridge Marsh and Parker River (*F = *244.8, *p *<* *0.001). Elevation also differed between coastal and inland sites (*F = *545.02, *p *<* *0.001), such that coastal sites had higher elevations (*x̄* = 1.54 ± 0.01 m) than inland sites ($\overset{¯}{x}$ = 1.21 ± 0.01 m). Fledged nests (*n* = 74) were located in areas of significantly higher marsh elevation ($\overset{¯}{x}$ = 138.3 ± 2 cm) than flooded nests (*n* = 32; $\overset{¯}{x}$ = 139.4 ± 3 cm; *GLMM,* χ^2^ = 13.79, *p *<* *0.001; Table 1; Appendix 2). We also found a significant difference in total nest height (surface elevation (cm) + bottom nest height (cm)) between fledged and flooded nests (*GLMM,* χ^2^ = 13.26, *p *<* *0.001), with fledged nests having significantly higher total nest height ($\overset{¯}{x}$ = 149.8 ± 2.1 cm) than flooded nests ($\overset{¯}{x}$ = 143.9 ± 3.1 cm).

### 3.2 Do females exhibit fidelity in their nest placement?

**Figure 5:**
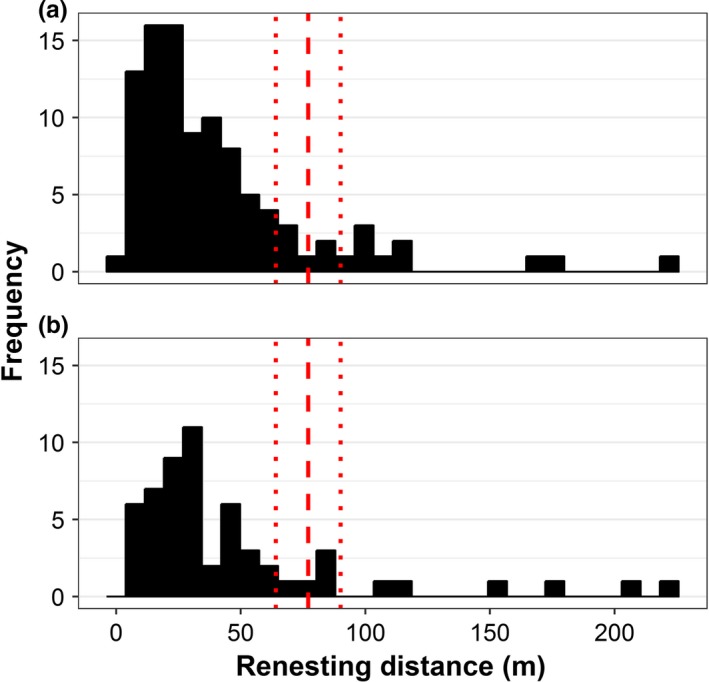
Frequency distribution of renesting distances (distances between subsequent nesting locations, measured across 1–5 repeat nesting attempts) of female saltmarsh sparrows (a) within years (*n* = 78 females, 98 pairs of nests) and (b) across years (*n* = 45 females, 57 pairs of nests). Dashed line indicates the 77 m diameter of average female home range core area, with dotted lines indicating the standard error (±13 m; Shriver et al., 2010)

Within and across breeding seasons, we found high fidelity in nesting location. Within a breeding season, 87% of females (*n* = 78) renested within the diameter of the average female core area (77 m); 5% of females moved 78 to 100 m, 6% moved 100 to 200 m, and 1% moved more than 200 m from a previous nest (Figure 5a). The mean renesting distance (distance between subsequent nesting attempts of the same female within a breeding season) was significantly less than the average home range core area diameter of 77 m ($\overset{¯}{x}$ = 40.5 m, *t *=* *−9.58, *p *<* *0.001). Across years, 85% of females (*n* = 45) renested within this core area distance; 5% returned to nest within 78 to 100 m, 7% renested between 100 and 200 m, and only 3% renested more than 200 m from the previous year's nest (Figure 5b). The mean renesting distance between years was significantly less than the average core area distance of 77 m ($\overset{¯}{x}$ = 47 m, *t *=* *−4.76, *p *<* *0.001).

### 3.3 Do females make changes in their nest site selection and structure based on the outcome of their prior nesting attempts?

**Figure 6:**
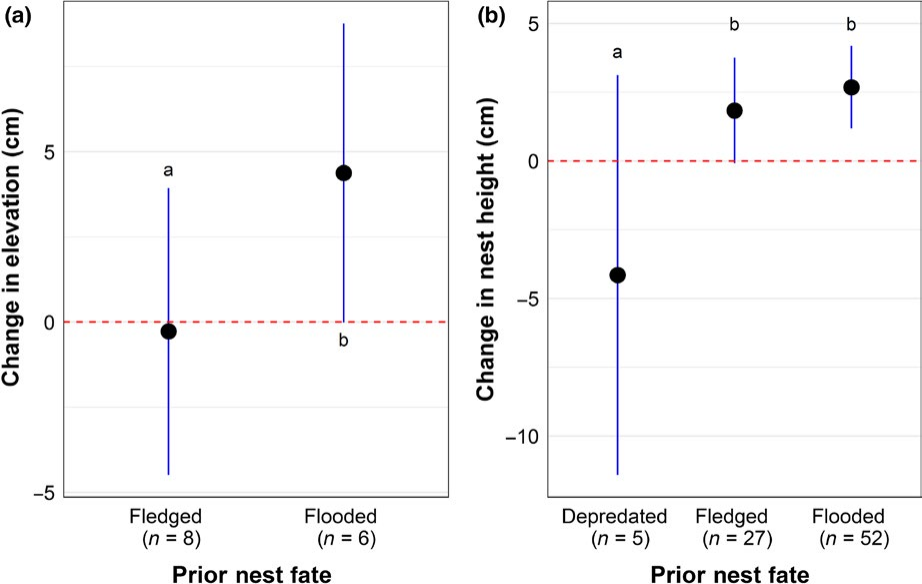
Changes in (a) elevation (± 95% CI) and (b) bottom height (±95% CI) of saltmarsh sparrow nests between subsequent nesting attempts of the same individual female according to first nest fate. Dashed line indicates no change in surface elevation between nesting attempts (depredated)

Nest characteristics differed between the first and second nesting attempts for females (*n* = 84 pairs of nests), according to the fate of their first nest (Table 2; Appendix 3). We found a significant difference in the change in marsh elevation for the renesting locations of females whose prior nest was successful and those that were flooded (*GLMM,* χ^2^ = 5.19*, p *=* *0.02; Figure 6a). Females for which the prior nest failed due to flooding chose a site with slightly higher elevation for their subsequent nest ($\overset{¯}{x}$ *= *2.7 ± 1.8 cm), while those that were successful in their previous attempt did not choose a location with a different elevation for their subsequent nest ($\overset{¯}{x}$ *= *−0.3 ± 1.5 cm). Changes in nest height (measured to the bottom of the nest cup) between nesting attempts also differed among females based on the outcome of the previous nesting attempt (*GLMM,* χ^2^ *= *6.77*, p *=* *0.03; Figure 6b). Females that experienced depredation decreased their nest height by 4.2 ± 2.3 cm and those that were flooded in their previous attempt increased nest height by 2.7 ± 0.8 cm (*Tukey*,* z *=* *−2.59, *p *=* *0.02). Individuals that failed due to flooding did not show a significant difference in nest height change compared to those that were successful (increased nest height by 1.8 ± 0.9 cm), and those that were depredated show a marginally significantly different change (*p* = 0.06) compared to those that were successful. Of the females that experienced flooding in the prior nesting attempt, 55% increased the bottom height of their second nesting attempt and were then successful. Similar trends were observed for lip nest height, but were all only marginally significant (*GLMM,* χ^2^ *= *5.22*, p *=* *0.07). We did not find any difference in the changes in canopy cover, vegetation composition, or cup depth between nesting attempts for any of the fate categories (Table 2, Appendix 3).

**Table 2:** Mean change ± SE in nest characteristics in renesting attempts for 64 females with 84 pairs of nests in each of the three prior fate categories—flooded, fledged, and depredated (fate category refers to the fate of the first nest attempt). Results of Tukey HSD comparisons provided, with different letters indicating statistically different comparisons

	Flooded (*n* = 52)		Fledged (*n* = 27)		Depredated (*n* = 5)	
Surface elevation (cm)^a^	2.7 ± 1.8	A	−0.3 ± 1.5	B	–	NA
Total Height (cm)^a^	3.1 ± 2.4	A	2.5 ± 1.2	A	–	NA
Bottom Nest Height (cm)	2.7 ± 0.8	A	1.8 ± 0.9	A	−4.2 ± 2.3	B
Lip Nest Height (cm)	2.7 ± 0.9	A	2.2 ± 1.0	A	−4.5 ± 4.8	A
High Marsh (%)	−5.7 ± 3.9	A	1.1 ± 7.7	A	−12.8 ± 36.0	A
Canopy Cover (%)	0.7 ± 4.3	A	−0.3 ± 6.2	A	−10.0 ± 23.1	A
Cup Depth (cm)	0.0 ± 0.4	A	0.1 ± 0.7	A	−0.3 ± 2.9	A
Renesting distance w/in years (m)^b^	42.5 ± 5.4	A	33.0 ± 5.0	AB	54.0 ± 6.4	B

a Sample size for elevation and total height (fledged, *n* = 8; flooded, *n* = 6).

b Sample size for renesting distance within years (*n* = 98 pairs of nests: flooded, *n* = 62; fledged, *n* = 29; depredated, *n* = 7).

**Figure 7:**
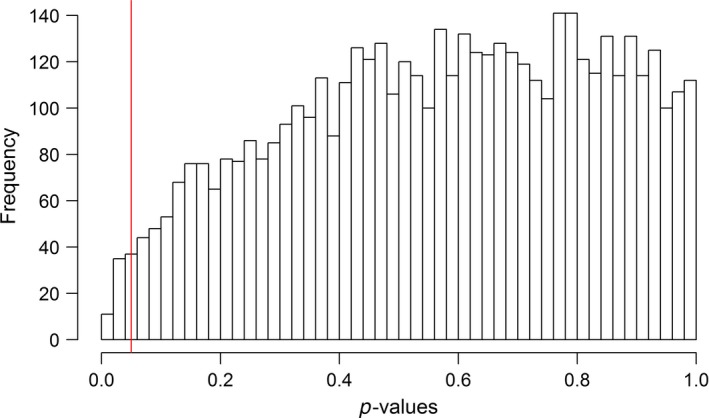
Frequency distribution of randomization test for a relationship of changes in nest height and prior nest fate. Results show the distribution of *p*‐values from 5000 models testing this relationship for nest height data with the first and second nesting attempts drawn in a randomized order. Red vertical line indicates *p* = 0.05

Results of the randomization test on the relationship of bottom nest height and prior nest fate revealed that 63 (1.26%) out of 5,000 randomized data models yielded a significant result (Figure 7), suggesting that the significant result in our observed nest data could not be explained by random chance alone. Although we also observed a significant relationship between the changes in nest elevation by prior nest fate, our sample size was too small to effectively test for the probability of the observed results occurring by random chance. Given the extremely low probability of females exhibiting changes in bottom nest height by random chance, it is likely that similar findings would occur with a larger sample size for nest elevation as well.

**Figure 8:**
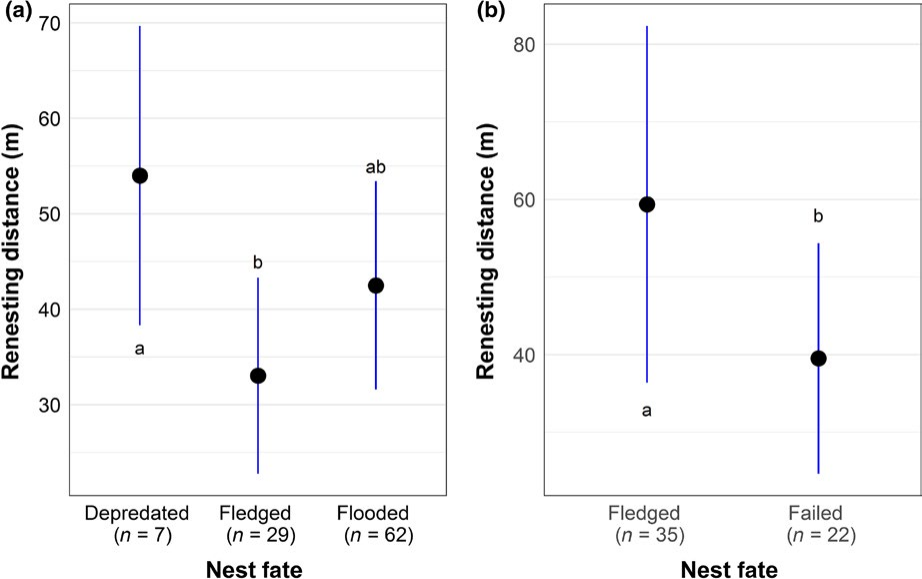
Distances (±95% CI) between locations of successive nesting attempts of the same individual saltmarsh sparrow female by fate of the previous nest. (a) Within a year; (b) across years

Within years, distances between subsequent nest locations of individual females ranged from 2 to 215 m between nesting attempts (98 pairs of nests from 78 females). Females renested an average of 33 m from their prior nesting attempts when successful, 42 m when flooded, and 53 m when depredated. The renesting distance differed significantly between females with fledged and depredated prior nesting attempts (*GLMM*,* F* = 3.02, *p *=* *0.05), but not between flooded and depredated prior nesting attempts (Table 2; Figure 8a). Over the 5 years of the study, we monitored 45 females with 57 paired nesting attempts across years, including one individual that was detected yearly from 2011 to 2014 (range = 2–6 nests/individual). Across years, the distance between nest locations ranged from 4 to 224 m. We found a trend for a larger between‐year renesting distance for females with prior nest failure (59 m) compared to those with successful nests (39.5 m, *GLMM,* χ^*2*^ = 3.11, *p *=* *0.08; Figure 8b); this pattern was not significant when evaluated across the three specific nest fates of fledged, flooded, or depredated.

## 4 DISCUSSION

Nest flooding and depredation risks are important selective factors that have shaped the evolution of reproductive strategies in marsh nesting birds (Greenberg et al., 2006; Picman, Milks, & Leptich, 1993). Saltmarsh sparrows have been associated with tidal marshes for several million years (Greenlaw, 1993); during this time, they have evolved strategies to mitigate flooding risks temporally, through rapid renesting following nest failure during peak inundation periods. It has been hypothesized that timing of nest initiation relative to the flood tides is the primary adaptive nesting strategy, rather than selection of nest sites that spatially minimize flooding risk (Gjerdrum et al., 2005; Shriver et al., 2007). Our findings suggest that nest characteristics, including height, canopy presence, and elevation within the marsh, may also influence nesting success and that females exhibit plasticity in nesting behavior, which may be important for balancing selective pressures in a dynamic environment.

We found that flooded, depredated, and successful saltmarsh sparrow nests differed in height, canopy presence, and marsh surface elevation. Specifically, successful nests were built higher in the vegetation, had more canopy cover, and were in higher elevation areas of the marsh than those that flooded. Several previous studies failed to find a consistent relationship between nest structures and nest success, despite strong evidence of nest site selection preferences for characteristics that confer resistance to flooding (Gjerdrum et al., 2005; Humphreys et al., 2007; Ruskin et al., 2015; Shriver et al., 2007). Our findings are consistent with these nest characteristics having a fitness effect, and the large sample size in our study (536 nests vs. 69 −160 nests in prior studies) may have allowed more power to detect small effect sizes. Furthermore, the high resolution of the RTK elevation measurements may have enhanced the power of our comparisons in elevation of nest locations. Another possible explanation may lie in the inclusion in our study of two inland, estuarine sites (Chapman's Landing and Lubberland Creek marshes, located in the Great Bay Estuary of New Hampshire), which have dampened tidal regimes compared to the coastal sites on which prior studies have taken place. Sparrows nesting on these marshes have been found to be among the most productive with among the lowest rates of nest flooding compared to other marshes in the species range (Ruskin et al., 2017a,b). It is possible that in these inland marshes, the adaptive (fitness) benefits of the nest site preferences are realized, whereas the extremely high rates of nest flooding experienced in coastal marshes today (which are increasing due to sea‐level rise; Bayard & Elphick, 2011) override any potential adaptive benefits of nest structure differences for the range of effect sizes observed.

Tidal height has strong effects on flooding probability, and successful nests withstand higher tide heights than those that fail due to flooding (Bayard & Elphick, 2011). It is intuitive that nests that are placed higher in the vegetation will be less impacted by tidal flooding than those placed lower, and damage from multiple flooding events may have cumulative impacts (Bayard & Elphick, 2011; Walsh et al., 2016). The height of the nest in relation to tidal height, however, is influenced both by nest height and by marsh surface elevation at the nest location. Elevation is known to influence nest site selection in the saltmarsh sparrow (DiQuinzio, Paton, & Eddleman, 2002; Gjerdrum et al., 2005) and other tidal marsh nesting species, such as clapper rail (*Rallus crepitans*; Valdes, Hunter, & Nibbelink, 2016), Nelson’s sparrow (*Ammospiza nelsoni subvirgatus*; Shriver et al., 2007), and willet (*Tringa semipamata*; Burger & Shisler, 1978), which build nests in areas of higher elevation compared to random locations on the marsh. Our finding that nest elevation differs between successful and flooded saltmarsh sparrow nests indicates that there are subtle elevational differences that influence nesting success within the preferred higher elevation areas of the marsh. A few centimeters in marsh elevation can make the difference between successful and flooded nests. When nest height and marsh surface elevation were taken additively together in the metric of total nest height, the difference in total nest height between successful and flooded nests in this study was 5.9 cm, remarkably consistent with the findings of Bayard and Elphick (2011) of a 6 cm difference in the maximum tide heights withstood by successful and flooded nests.

While successful nests were placed higher in the vegetation than those that flooded, they were also lower than those that were depredated, supporting the idea that there is a trade‐off between predation and flooding (Greenberg et al., 2006; Ruskin et al., 2017a). A trade‐off between predation and flooding along a gradient of nest height also occurs in the closely related seaside sparrow (*Ammospiza maritimus*). Hunter et al. (2016) found that nests located higher in the vegetation had a greater probability of predation and lower probability of flooding, while those located lower in the vegetation had a lower predation probability and higher flooding probability. While optimal nest height may entail a trade‐off between predation and flooding, the presence of a nest canopy may confer advantages against both threats, by providing a structure that prevents egg loss while also providing concealment (Humphreys et al., 2007). Our results were consistent with nest canopy providing protection against egg loss, as successful nests had more of a canopy presence than flooded nests (although this difference was not statistically significant when modeled using percent canopy cover rather than categorical presence of canopy, likely due to inconsistencies in observers’ visual estimates of percentages in the field). Our results did not provide clear support for the role of canopy in providing protection from predation, as successful and depredated nests did not differ significantly in canopy cover (although there was an apparent nonsignificant trend for less canopy presence in depredated nests), nor did females with depredated nests increase the amount of canopy cover in their subsequent renesting attempt. The latter may at least in part be due to relatively small sample sizes for depredated nests, as flooding is the far greater source of nest failure in our study marshes. Additionally, while nest canopies may be beneficial against avian predators that use visual cues (e.g., crows, gulls), they may be less advantageous in protecting nests against mammalian predators (e.g., mesocarnivores), which rely strongly on olfactory cues. In our study marshes, the presence of both avian and mammalian predators may confound the expected association between nesting success and canopy cover for depredated vs. successful nests.

Making repairs or changes to nest structure or placement increases the likelihood of success in tidal marsh nesting birds (Beckmann et al., 2015; Burger, 1979). We found female saltmarsh sparrows altered their nest placement and structure in subsequent nesting attempts based on the fate of their previous nest. Behavioral plasticity, via adjustments to nest structure and site selection based on immediate environmental conditions, may be important mechanisms for species persistence in the dynamic tidal marsh environment (Refsnider & Janzen, 2012). By exhibiting plasticity in nesting behavior following a cause‐specific nest failure, saltmarsh sparrows may be able to respond to the selective pressure that is stronger at a given time or place, given variation in predation and flooding risks (Ruskin et al., 2017a). We found differential changes in nest height and elevation following a failure due to flooding or predation. Specifically, females that experienced nest flooding increased the height of their nest and placed their nests in higher elevation locations of the marsh in successive attempts, thereby adopting behaviors to mitigate flooding, while those that were predated decreased the height of their nest, consistent with minimizing visibility to predators. Females that experienced nest flooding also had a slightly lower proportion of high marsh vegetation in their subsequent nests, and successful nests had ~5% lower high marsh composition than flooded nests, although these trends were not statistically significant. High marsh vegetation is relatively simple in structure; nests constructed with a mixture of *S. patens* and *S. alterniflora* may have greater structural support, may have better withstand flooding, and may be more able to retain overall nest shape during and following flooding events than nests comprised of primarily *S. patens* (Walsh et al., 2016). The effect of vegetation composition, however, must be balanced with surface elevational differences in high vs. low marsh, which likely explains the slight differences that were observed (~6% change in vegetation composition, co‐occurring with a slight increase in surface elevation).

Nesting plasticity has been found in two other studies of tidal marsh birds. DiQuinzio et al. (2002) found that female saltmarsh sparrows made changes in nest height and vegetation composition following restoration of a tidally restricted marsh, despite no changes in marsh surface elevation. The observed changes in nest height and switch in vegetation composition from *Phragmites australis* to *S. patens*,* S. alterniflora*, and *D. spicata* occurred in the year immediately following tidal restoration, suggesting that saltmarsh sparrows exhibit plasticity in their nest vegetation selection and are able to respond to moderate habitat alteration over a rapid timescale. Hunter et al. (2016) found plasticity in nesting behavior of seaside sparrows in response to variably predictable threat risks. Seaside sparrows nested at lower height in years with high predation risks, but increased nest height following failure due to flooding in years with unpredictable tidal flooding caused by wind events (Hunter et al. 2016).

Plasticity in nesting behavior can also take the form of shifts in habitat selection to areas with a different vegetation composition or different risk of threat, for example, predation (Chalfoun & Martin, 2010). Here, we found support for our hypothesis that females that experienced predation in their previous nesting attempt renested at a greater distance than those that were successful or flooded. By renesting farther from a previous nesting attempt, a female may be able to find an area with lower predator densities and different vegetation composition, such as taller vegetation or different species, which may increase concealment. In contrast, it may be more beneficial for females that experience nest flooding to renest near their previous nest and make structural changes rather than to renest in a different location, if timing of reproduction in relation to the tidal cycle and nest structure is generally more important than nest placement within the marsh (Shriver et al., 2007).

Females are faced with nest site selection trade‐offs across seasons as well. With a limited nesting window, it may be more advantageous for females to spend less time scouting for new nesting locations upon arrival on the breeding grounds and quickly begin nesting using information gained from prior nesting experiences. This informed nest site fidelity would allow them to benefit from awareness of local environmental factors such as food abundance, tidal regime, or predation pressure (Chalfoun & Schmidt, 2012; Switzer, 1997), and, on a finer scale, elevation differences and vegetation patterns. Across years, 85% of saltmarsh sparrow females in this study returned to nest within their previous home range core area, with some renesting within a few meters of a previous nest. This high degree of nest placement fidelity may be informed by prior success in relation to flooding risk, predation pressure, and accessibility to mating opportunities. Informed site fidelity may also confer reproductive advantages in this highly promiscuous mating system (Hill et al., 2010), if mate accessibility varies spatially across the marsh.

The rapid rate of global climate change likely limits adaptive genetic changes at a population level (Berteaux, Reale, McAdam, & Boutin, 2004; Refsnider & Janzen, 2012). Mechanisms occurring at the individual level, however, such as behavioral plasticity, may provide some capacity for adapting to novel environmental effects (Refsnider & Janzen, 2012). Plasticity in nesting behavior of saltmarsh sparrows may allow them to quickly adapt to modest changes in tidal regime, habitat loss, and fragmentation. This plasticity, however, is likely insufficient in the face of sea‐level rise, which reduces high marsh habitat and modifies tidal regimes that disrupt synchronous breeding of sparrows with the 28‐day tidal cycle. The direct impacts of sea‐level rise are predicted to reduce the reproductive success of saltmarsh sparrows (Bayard & Elphick, 2011), which have already declined at a rate of 9% annually from 1998 to 2012 (Correll et al., 2016), leaving the species vulnerable to extinction within the next 50 years (Field et al., 2016, 2017). The apparent adaptive capacity of saltmarsh sparrows, however, may enhance their ability to respond to management interventions targeted to mitigate nest flooding. On the other hand, the high degree of nest site fidelity that we observed in this study raises questions about how readily sparrows will respond to marsh restoration, such as large‐scale thin level sediment deposition, which would make nesting habitat unavailable for one or more breeding seasons. This would likely require sparrows to move to a new nesting location, potentially several hundred meters or more away from the location to which they have shown fidelity. Accordingly, the potential benefits and limits of sparrow's nesting plasticity should be evaluated in future restoration efforts.

## CONFLICT OF INTEREST

None declared.

## AUTHOR CONTRIBUTIONS

BB and AIK designed the study, with help from KMO and JW; BB and JW collected field data; BB analyzed the data, with guidance from AIK and JW; KMO provided logistical support; BB wrote the manuscript with substantial input from AIK; all authors edited the manuscript.

## DATA ACCESSIBILITY

Data used in this publication, including nest structural features and fates, changes in these features and renesting distances between subsequent nesting attempts, are archived in DRYAD https://doi.org/10.5061/dryad.q64b0n2.

## Appendix 1 Total number of saltmarsh sparrow nests with assigned fates for four study sites and five years. Percentage of nests for which the female associated with the nest was captured is also shown.

### 1.1

**Table:**

Site	2011	2012	2013	2014	2015	Total nests found	Percentage of females captured
Chapman's Landing	45	52	60	41	39	237	82.7
Eldridge Marsh	35	33	30	18	32	148	65.5
Lubberland Creek	–	15	19	13	20	67	65.7
Parker River NWR	–	–	28	34	22	84	66.7
Total Nests	80	100	137	106	113	536	73.3

## Appendix 2 Results of generalized linear mixed models (GLMMs) testing for differences in features of 536 saltmarsh sparrow nests across the fates of fledged, flooded, and depredated. Location refers to inland/coastal sites, and site refers to the four separate study marshes.

### 2.1

**Table:**

Nest characteristic	Model fixed effects	Test	Test stat	Test stat value	DF	*p*‐value
Bottom height (cm)	Nest fate + location	GLMM	Chi‐sq	28.06	2	<0.001
	fledged–depredated	Tukey	*z*‐value	−2.02		0.10
	flooded–depredated	Tukey	*z*‐value	−4.51		<0.001
	flooded–fledged	Tukey	*z*‐value	−3.98		<0.001
Lip height (cm)	Nest fate	GLMM	Chi‐sq	29.11	2	<0.001
	fledged–depredated	Tukey	*z*‐value	−2.19		0.07
	flooded–depredated	Tukey	*z*‐value	−4.69		<0.001
	flooded–fledged	Tukey	*z*‐value	−4.06		<0.001
Canopy cover (%)	Nest fate	GLMM	Chi‐sq	0.16	2	0.92
Cup depth	Nest fate	GLMM	Chi‐sq	2.69	2	0.26
High marsh (%)	Nest fate	GLMM	Chi‐sq	6.81	2	0.03
	fledged–depredated	Tukey	*z*‐value	−2.04		0.09
	flooded–depredated	Tukey	*z*‐value	−0.72		0.75
	Flooded–fledged	Tukey	*z*‐value	2.15		0.08
Elevation (cm)^a^	Nest fate + Site	GLMM	Chi‐sq	13.79	1	<0.001
Total Height (cm)^a^	Nest fate + Site	GLMM	Chi‐sq	13.26	1	<0.001
Canopy presence (coded; −1 no canopy, 0 partial, 1 complete)	Nest fate + location	GLMM	Chi‐sq	10.11	2	0.006
	Fledged–depredated		*z*‐value	1.177		0.46
	Flooded–depredated		*z*‐value	−0.789		0.7
	Flooded–fledged		*z*‐value	−3.175		0.004

## Appendix 3 Results of generalized linear mixed models for changes in characteristics of saltmarsh sparrow nests between nesting attempts (*n* = 84 pairs of nests), with respect to the first nest fates of fledged, flooded, and depredated. * denotes significance at *p* < 0.05, † indicates marginally significant.

### 3.1

**Table:**

Change response	Effects	Test	Test stat	Test stat value	DF	*p*‐value
Bottom height (cm)	Prior nest fate	GLMM	Chi‐square	6.77	2	0.03*
	Flooded–fledged	Tukey	*z*‐value	0.69		0.75
	Depredated–fledged	Tukey	*z*‐value	2.2		0.06†
	Depredated–flooded	Tukey	*z*‐value	‐2.59		0.02*
Lip height (cm)	Prior nest fate	GLMM	Chi‐square	5.23	2	0.07†
	Flooded–fledged	Tukey	*z*‐value	0.34		0.94
	Depredated–fledged	Tukey	*z*‐value	‐2.07		0.08†
	Depredated–flooded	Tukey	*z*‐value	‐2.29		0.05*
Canopy cover (%)	Prior nest fate	GLMM	Chi‐square	0.79	2	0.67
Cup depth	Prior nest fate	GLMM	Chi‐square	0.045	2	0.98
High marsh (%)	Prior nest fate	GLMM	Chi‐square	0.87	2	0.65
Elevation (cm)^a^	Prior nest fate	GLMM	Chi‐square	3.62	1	0.05*
Total height (cm)^a^	Prior nest fate	GLMM	Chi‐square	0.25	1	0.61
